# A tale of sugars’ tails—diverse acyl chains decorate sugars in *Solanum* species’ trichomes

**DOI:** 10.1093/plphys/kiae345

**Published:** 2024-06-14

**Authors:** Lara Pereira

**Affiliations:** Assistant Features Editor, Plant Physiology, American Society of Plant Biologists; Ecology and Evolutionary Biology, School of Biosciences, University of Sheffield, Sheffield S10 2TN, UK

Collectively, plants synthesize hundreds of thousands of small organic compounds. Only a minority of them belong to primary metabolism, defined by being essential and common to all plants. The vast majority are specialized metabolites, which are structurally diverse, usually limited to certain taxonomic groups, and produced in specific tissues and under specific conditions. These specialized metabolites contribute to plant adaptation by playing a myriad of roles throughout a plant's life, from recruiting pollinators by conferring a flower scent (Huang and Dudareva 2023), to the modulation of root microbiota by secreting a specific combination of metabolites (Huang et al. 2019). One of the most relevant functions of specialized metabolism is plant defense—this chemical defense includes direct interactions, such as the suppression of oviposition, and indirect interactions, such as the attraction of natural enemies of attacking herbivores (Huang and Dudareva 2023).

Acylsugars are one group of such compounds produced across the Solanaceae family, among others (Moghe et al. 2023). These trichome-specific metabolites, composed by a sugar core decorated with several acyl chains, are exuded in the form of a sticky extract with defensive properties. Even though acylsugars are synthesized from basic, simple units present in primary metabolism, they present a remarkable structural diversity, with variations in sugar core composition, acyl chain length, and branching patterning. The genus *Solanum*, which includes more than 1,300 species distributed in 12 major clades, is a great system to investigate the evolution of acylsugars biosynthesis (Särkinen et al. 2013). However, research focused on species belonging to the well-known Potato clade, which includes the horticultural crops tomato and potato, while other clades remain mostly unexplored.

In the current issue of *Plant Physiology*, Fiesel et al. (2024) used an impressive combination of analytical chemistry and molecular biology techniques to characterize acylsugar chemical diversity within the underexploited clades of *Solanum* genus, with a focus on brinjal eggplant (*Solanum melongena*), and to functionally validate an enzyme of biosynthetic pathway producing these compounds.

Initially, the authors analyzed surface metabolite extracts from brinjal eggplant using liquid and gas chromatography coupled with mass spectrometry and further resolved their structures by nuclear magnetic resonance. They annotated 38 acylsugars, consisting of 16 acylhexoses and 22 acyldisaccharides. Interestingly, the acylhexose and acyldisaccharide sugar cores are *myo*-inositol and 4-O-β-arabinopyranosyl *myo*-inositol, respectively, in contrast with the sucrose-based acylsugars found in cultivated tomato (*Solanum lycopersicum*) (Fan et al. 2016). In addition, they identified hydroxylated C12, C14, and C16 acyl chains not previously reported in Solanaceae acylsugars.

Later, capitalizing on the detailed and precise annotation of brinjal eggplant acylsugars, the authors used a simplified approach to survey the leaf surface extracts acylsugar diversity from 31 *Solanum* species outside of the Potato clade (Fig.). The identified compounds varied in acyl chain lengths, functional groups, and combinations of chain types and positions—including C2 to C18 acyl chains in saturated, unsaturated, and hydroxylated forms. The hydroxylated acyl chains are especially interesting because they may act as “chemical handles” that can be further modified. In fact, glycosylated acyl chains were founded in 3 of the analyzed species. Furthermore, 2 of the studied species present a sticky fruit surface, and, interestingly, more than 100 fruit-specific acylsugars were identified by analyzing fruit surface extracts.

**Figure.**
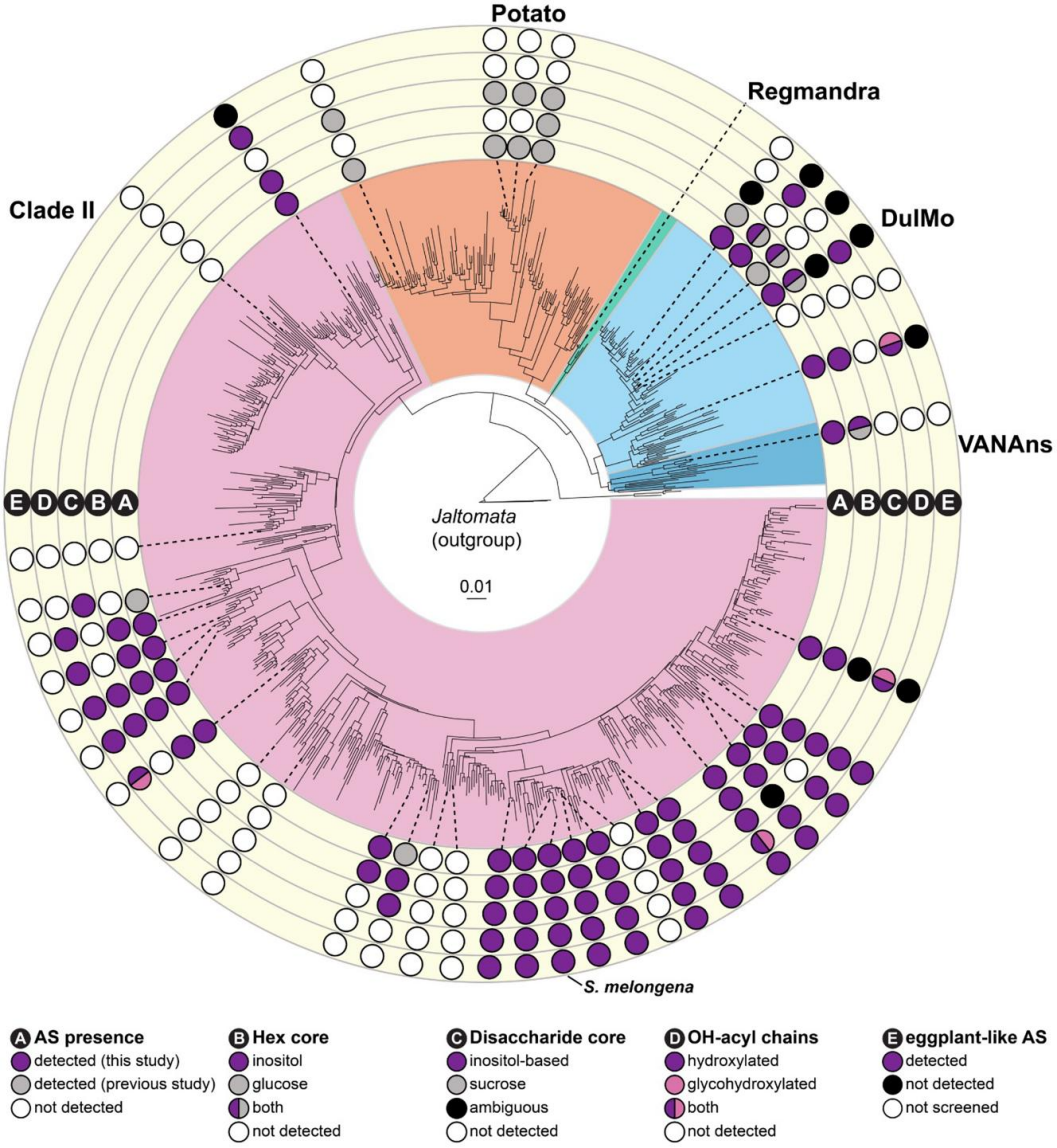
Distribution of acylsugar traits across the *Solanum* genus (from Fiesel et al. 2024). Five rings (labeled A–E) surrounding the phylogeny represent different acylsugar traits that vary across Solanum species. **A)** AS presence: presence of detectable acylsugars in aerial surface (i.e. trichome) extracts. **B)** Hex core: identity of hexose sugar core in detected acylhexoses. **C)** Disaccharide core: identity of disaccharide sugar core in detected acyldisaccharides. **D)** OH-acyl chains: identity of unusual acyl chains bearing hydroxyl or glycosylated hydroxyl groups in detected acylsugars. **E)** Eggplant-like AS: presence of detectable acylsugars with acyl chain and sugar core composition identical to brinjal eggplant acylsugars.

To uncover the molecular basis of acylsugar biosynthesis within the Clade II *Solanum* species, the authors used a transcriptomics approach to find differentially expressed genes between eggplant and *S. aethiopicum*. The latter one did not accumulate the most abundant acylsugar found in eggplant, and the authors hypothesized that this metabolic difference was caused by differential expression of the responsible acetylating enzyme. They searched for genes encoding for BAHD acyltransferases, a family of enzymes well known for their roles in plant specialized metabolism (D’Auria 2006). In vitro assays in *Escherichia coli* revealed that only 1 of the 7 tested enzymes was capable of adding and removing the acyl chain when using the target compounds as substrates.

Acylinositols reported in Fiesel et al. (2024) are likely synthesized through different pathways from the acylsucroses found in tomato. Interestingly, the biosynthetic pathway described in tomato for acylsugars is clustered in a so-called biosynthetic gene cluster—the genes encoding for the enzymes in the pathway are located next to each other in the genome and tightly regulated to be coexpressed in the same cells and under the same conditions (Fan et al. 2020; Kerwin et al. 2024). The characterization of a novel biosynthetic enzyme in eggplant within the acylinositol pathway could potentially lead to the discovery of a new biosynthetic gene cluster, offering a broader perspective of the evolutionary origin of these gene clusters.

The authors brilliantly decoded the chemical basis of this sticky exudate accumulated in leaf and fruit surfaces of several diverse *Solanum* species. However, what is the ecological relevance of these findings? One of the main functions of these mixtures of acylsugars is to immobilize or suffocate insects when they feed from plant tissues, and the effectivity of this interaction would probably depend on the absolute amount of acylsugars rather than their diversity (Moghe et al. 2023). Yet, other defense strategies are also promoted by acylsugars. For example, volatile acyl chains are released in the gut of larvae after feeding on trichomes, informing nearby predator ants (Weinhold and Baldwin 2011)—different predators respond to different acyl chains; therefore, in this case, structural diversity has adaptive significance. Acylsugars interfere in tritrophic interactions, deter oviposition, increase drought tolerance, and even promote plant-to-plant communication (Moghe et al. 2023). Different combinations of acylsugars may offer specific advantages under certain conditions, and this work paves the way to investigate the ecological functions of these versatile metabolites and their potential applications in crop breeding.
